# Supplementary material for: Accelerating hybrid XOR–CNF Boolean satisfiability problems natively with in-memory computing
Source: Nat Commun. 2026 Feb 19;17:2922. doi: 10.1038/s41467-026-69465-2 (PMC13031946; doi:10.1038/s41467-026-69465-2)
Supplement: Supplementary file 1 — Supplementary Information [file 41467_2026_69465_MOESM1_ESM.pdf]

Supplementary Information for "Accelerating Hybrid XOR–CNF Boolean Satisfiability Problems Natively with In-Memory Computing"

Haesol Im,[1,†] Fabian Böhm,[2,†] Giacomo Pedretti,[3] Noriyuki Kushida,[1] Moslem Noori,[1] Elisabetta Valiante,[1] Xiangyi Zhang,[1] Chan-Woo Yang,[1] Tinish Bhattacharya,[4] Xia Sheng,[3] Jim Ignowski,[3] Arne Heittmann,[5] John Paul Strachan,[5,6] Masoud Mohseni,[3] Raymond Beausoleil,[3] Thomas Van Vaerenbergh,[2] and Ignacio Rozada[1,*]

[1]*1QB Information Technologies (1QBit), Vancouver, BC, Canada*
[2]*HPE Labs, Hewlett Packard Enterprise, Brussels, Belgium*
[3]*HPE Labs, Hewlett Packard Enterprise, Milpitas, CA, USA*
[4]*University of California, Santa Barbara, CA, USA*
[5]*Peter Grünberg Institute (PGI-14), Forschungszentrum Jülich GmbH, Jülich, Germany*
[6]*RWTH Aachen University, Aachen, Germany*

SUPPLEMENTARY NOTE 1: PERFORMANCE OF WALKSAT-XNF ACCELERATOR FOR DIFFERENT PROBLEM REPRESENTATIONS

In Supplementary Figure 1, we show the relative area advantage, energy per iteration, time-to-solution (TTS) and energy-to-solution (ETS) for the simulations of the 28 nm RRAM WalkSAT-XNF accelerator for different problem representations (CNF, CNF-PP, XNF, and XNF-PP). For the relative crossbar array area advantage, we compare the area against the CNF representation and find that the smallest advantage is attained for the CNF-PP representation ($(2.4 \pm 1.5)\times$), followed by XNF ($(7.8 \pm 4.3)\times$) and XNF-PP ($(26.6 \pm 13.1)\times$). For the energy per iteration, the highest value is for the CNF representation (median: $1.3 \cdot 10^{-10}$ J, IQR: $9.9 \cdot 10^{-11}$ J), followed by XNF (median: $1.2 \cdot 10^{-10}$ J, IQR: $1.9 \cdot 10^{-11}$ J), CNF-PP (median: $1.1 \cdot 10^{-10}$ J, IQR: $1.2 \cdot 10^{-11}$ J), and XNF-PP (median: $3.6 \cdot 10^{-11}$ J, IQR: $4.6 \cdot 10^{-11}$ J). Median TTS and ETS, as well as the percentage of solved instances, are summarized for the different problem representations in Supplementary Table 1. Across all problem representations, CNF exhibits the worst performance, followed by XNF, CNF-PP, and XNF-PP. Notably, WalkSAT-XNF is able to find all solutions only in the XNF and XNF-PP representations.

* Corresponding author: ignacio.rozada@1qbit.com

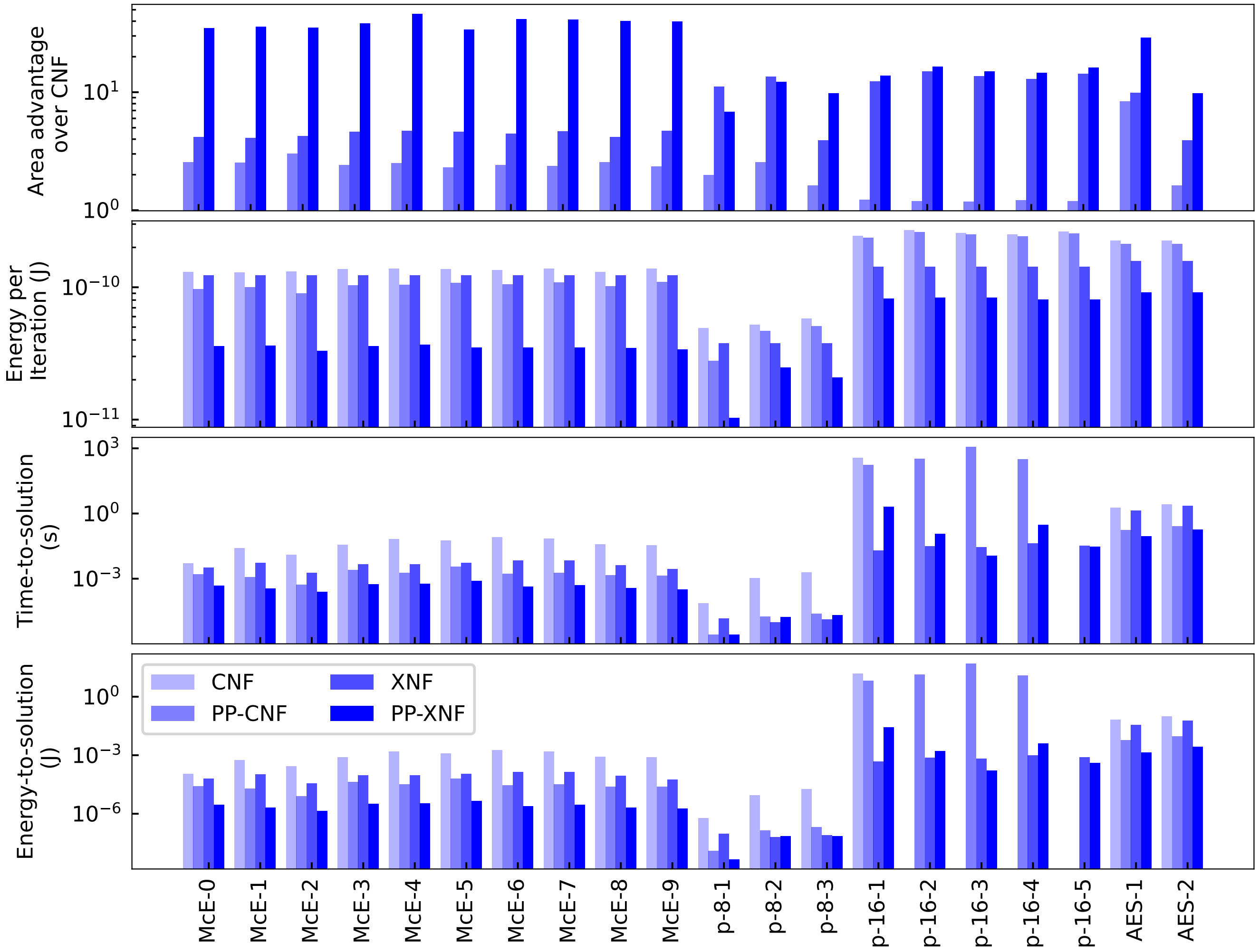

Supplementary Figure 1: **Comparison of area, energy and computation time for different problem representations** Relative area advantage, energy per iteration, time-to-solution and energy-to-solution for the benchmarking instances for different problem representations (CNF, CNF-PP, XNF, and XNF-PP). Data is not shown in cases where WalkSAT-XNF was unable to find a solution.

(a)	CNF			CNF-PP		
	**TTS** (s)	**ETS** (J)	**Solved** (%)	**TTS** (s)	**ETS** (J)	**Solved** (%)
McEliece	0.04 (0.04)	$8.3 \cdot 10^{-4}$ $(8.7 \cdot 10^{-4})$	100	0.0016 (0.0005)	$2.8 \cdot 10^{-5}$ $(8.2 \cdot 10^{-6})$	100
MDP	$3.0 \cdot 10^{8}$ $(6.0 \cdot 10^{8})$	$1.3 \cdot 10^{7}$ $(2.6 \cdot 10^{7})$	50	239.1 (537.2)	9.6 (22.9)	87.5
AES	2.2 (0.4)	0.08 (0.01)	100	0.22 (0.05)	0.01 (0.002)	100
all	0.06 (94.8)	0.001 (3.9)	80	0.002 (42.1)	$3.3 \cdot 10^{-5}$ (1.7)	95

(b)	XNF			XNF-PP		
	**TTS** (s)	**ETS** (J)	**Solved** (%)	**TTS** (s)	**ETS** (J)	**Solved** (%)
McEliece	0.005 (0.002)	$9.6 \cdot 10^{-5}$ $(3.9 \cdot 10^{-5})$	100	$4.6 \cdot 10^{-4}$ $(1.8 \cdot 10^{-4})$	$2.7 \cdot 10^{-6}$ $(1.1 \cdot 10^{-6})$	100
MDP	0.02 (0.03)	$5.7 \cdot 10^{-4}$ $(7.5 \cdot 10^{-4})$	100	0.02 (0.16)	$2.8 \cdot 10^{-4}$ (0.002)	100
AES	1.8 (0.5)	0.05 (0.01)	100	0.14 (0.05)	0.002 $(7.1 \cdot 10^{-4})$	100
all	0.005 (0.03)	$1.1 \cdot 10^{-4}$ $(6.1 \cdot 10^{-4})$	100	$5.3 \cdot 10^{-4}$ (0.04)	$3.1 \cdot 10^{-6}$ $(6.4 \cdot 10^{-4})$	100

Supplementary Table 1: Median time-to-solution (TTS) and energy-to-solution (ETS), as well as percentage of solved instances, for the WalkSAT-XNF accelerator when solving the benchmarking instances in (a) CNF and CNF-PP representations, as well as in (b) XNF and XNF-PP representations. The IQR is shown in parentheses.

We analyze the relationship between the metrics in Supplementary Figure 1, clause degrees, instance sizes, and the hardness of solving instances in the XNF and XNF-PP representations. The ranges of variable and clause counts for the benchmarking instances before and after the conversions are summarized in Supplementary Figure 2. Problem

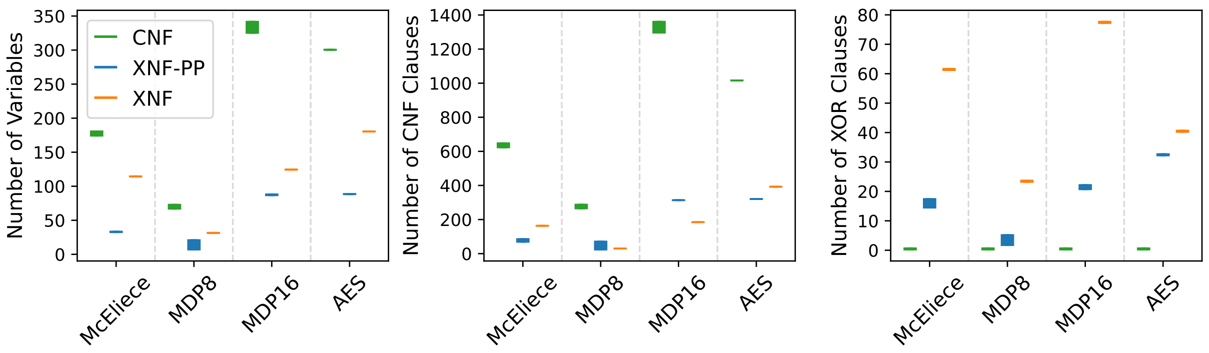

Supplementary Figure 2: **Variable and clause ranges before and after conversions**

size is the dominant factor that influences hardness, as is evident from the plots relating energy-per-iterations and instance size in Supplementary Figure 3a, and ITS and instance size in Supplementary Figure 3b. In Supplementary Figure 3c, we observe a positive correlation between the degrees of clauses and ITS. Specifically, higher XOR clause degrees tend to increase problem hardness, leading to larger ITS values. This observation aligns with empirical findings in Ref. [1], which uses random instances solved with CryptoMiniSat. However, it is important to note that this is not an entirely direct comparison, as our benchmarking experiments are based on structured problem instances, whereas the results in Ref. [1] are based on random instances under controlled settings. Additionally, we observe that the degrees of XOR clauses in the XNF-PP representation tend to be greater than those in the XNF representation. The increase in degree with respect to the CNF clauses is also observed after preprocessing, yet the degrees of the CNF clauses appear to have a weaker correlation with ITS.

We now compare the average TTS grouped by instance class and formulation class, along with their improvements, measured for both the CNF and XNF representations. The TTS metric TTS (CPU) is measured on a CPU-based implementation of WalkSAT-XNF, written in the Rust programming language. The TTS metric TTS (IMC) is given by the product of ITS and a latency of 6 ns, as shown in Supplementary Table 2. As shown in the Speedup columns,

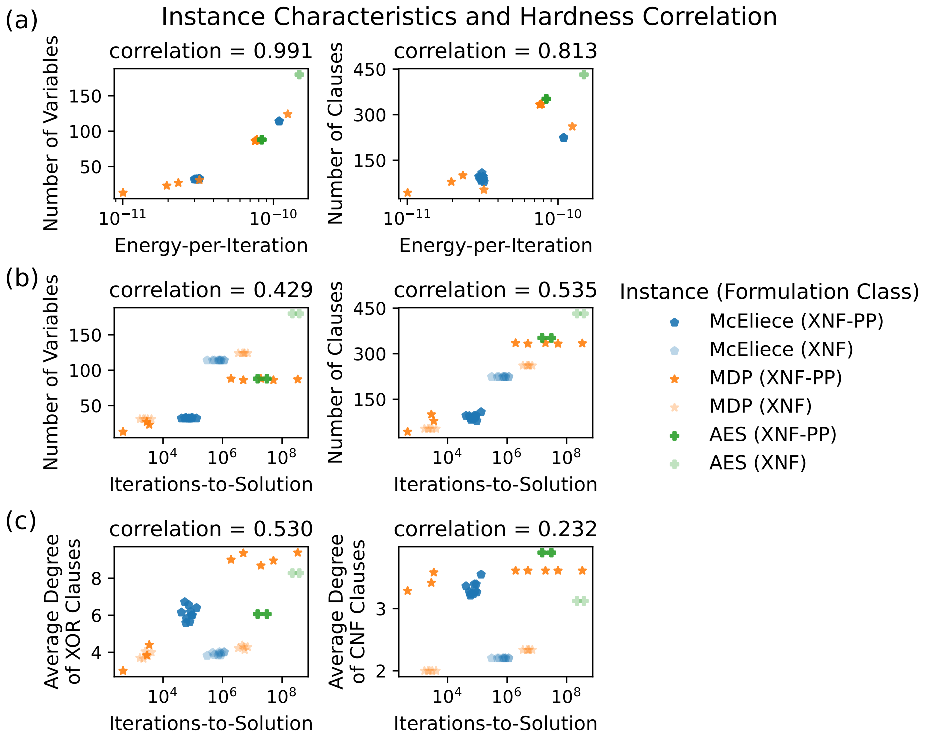

Supplementary Figure 3: **Relationship between problem size, clause degrees, and iterations-to-solution in XNF and XNF-PP representations** (a) Energy-per-iteration versus instance size. (b) ITS versus instance size. (c) ITS versus average clause degree.

the TTS measured with the IMC hardware shows an orders-of-magnitude improvement.

Instance Class	CNF-PP			XNF-PP		
	TTS (CPU)	TTS (IMC)	Speedup	TTS (CPU)	TTS (IMC)	Speedup
McEliece	0.15	0.0018	84	0.03	0.0005	73
MDP	352,646.54	282.7887	1,247	84.98	0.3104	274
AES	28.10	0.2178	129	26.19	0.1377	190
all	129,925.45	104.21	1,247	36.63	0.14	265

Supplementary Table 2: Comparison of average time-to-solution (TTS) achieved by CPU and IMC implementations across instance classes using WalkSAT-XNF.

SUPPLEMENTARY NOTE 2: STATE-OF-THE-ART SOLVERS INVOLVING XOR OPERATIONS

In this section, we list state-of-the-art solvers that provide native treatments for XOR clauses. xnfSAT [2] is a stochastic local search algorithm that extends the YalSAT solver [3] by adding support for XOR clauses. In YalSAT, a variable to be flipped is selected proportional to an exponential decay function, where the exponent is a weighted version of the break values. A break value is computed over a set of clauses becoming unsatisfied after the variable is flipped. xnfSAT constructs weights depending on the clause types. For CNF clauses, the weights are constructed as a function of the length of a CNF clause. For all XOR clauses, a fixed weight is used. Similar to WalkSAT-XNF, xnfSAT accounts for the fact that every literal in a satisfied XOR clause renders the clauses unsatisfied upon variable flips. This makes local variable flips that preserve XOR satisfiability computationally more complex than flipping variables within CNF clauses. It is worth noting that the IMC architecture within WalkSAT-XNF supports parallelized break value computations, offering potential speedups over the sequential break value computations used in xnfSAT. xnfSAT outperforms a CDCL solver when finding solutions to Boolean Brent equations [4].

CryptoMiniSat [5] is a CDCL-based solver originally designed for cryptographic problems, and a complete and

backtracking-based algorithm. The work Ref. [5] highlights the advantages of its native treatment of XOR clauses by treating them as a set of linear equations alongside CNF clauses. Working with a linear system, the solver is able to utilize Gaussian elimination on the system of XOR equations, and use the system for unit propagation, thereby reducing the search space. However, frequent Gaussian elimination can deter the solver's performance, as this operation scales as $O(n^x)$, with $x \in (2, 3)$, where n is the number of variables. Hence, restricting the Gaussian eliminations at the top levels within the search tree has been proposed. Due to its XOR handling capabilities, CryptoMiniSat can be used for applications ranging from cryptoanalysis to hash-based approximate model counting [6, 7].

The solver 2-Xornado [8] is based on the Davis–Putnam–Logemann–Loveland (DPLL) algorithm tailored to XOR-OR-AND normal form, where each clause is a disjunction of at most two linerals. A lineral is defined as the XORs of literals, thus serving as a generalization of a literal. 2-Xornado can treat the XOR clauses natively by treating them as 1-lineral clauses. One noted challenge is that long linerals tend to make the vertices of the implication graph more costly to propagate. To use 2-Xornado, XOR–CNF formulae can be transformed into the 2-XOR-OR-AND normal form by introducing auxiliary variables. This involves transforming CNF clauses into 2-literal clauses. In short, a degree-k CNF clause is reduced to $(k-2)$ 3-literal clauses with $k-3$ auxiliary variables. Each 3-literal clause is then converted to a set of 2-literal clauses with the addition of auxiliary variables. However, this transformation does not guarantee satisfiability of the resulting 2-literal clauses, since there is no known satisfiability-preserving reduction. This fact highlights the importance of selecting solvers that align with the structural characteristics of a given problem. The study Ref. [8] exhibits strong performance in solving ASCON cryptographic problems since the 2-XOR-OR-AND normal form offers a compact representation of the ASCON cryptosystem's algebraic structure given in algebraic normal form (ANF). The solver WDSat [9] is another DPLL-based algorithm that supports instances natively given in ANF. The branching technique within its search tree is inspired by the idea of the minimum vertex cover problem. The study also discusses the overhead of converting the ANF to XOR–CNF formulae, and the potential cost incurred by solvers that rely on Gaussian elimination to process XOR clauses.

CryptoMiniSat and xnfSAT can handle instances using XOR–CNF formulae directly, without requiring further translation, whereas 2-Xornado is suited for 2-lineral clauses and WDSat can be effectively used for problems given in ANF. Consequently, we proceed with CryptoMiniSat and xnfSAT as benchmarking solvers, as solving the problem in a representation close to its native form aligns with the objective of our work.

SUPPLEMENTARY NOTE 3: STATE-OF-THE-ART SAT HARDWARE ACCELERATORS

Various hardware accelerators have been proposed and demonstrated that solve SAT problems, including accelerators based on IMC [10–14] and Ising machines [15]. Supplementary Table 3 shows a comparison of current state-of-the-art SAT accelerators. Except for the Ising machine presented in Ref. [15], which requires a translation into quadratic unconstrained cost functions, all accelerators listed in Supplementary Table 3 implement and solve SAT problems formulated in CNF representation using stochastic local search (SLS) heuristics. Notably, while none of these accelerator devices support XOR clauses, it is still possible for them to solve hybrid XOR–CNF problems by mapping them to a CNF representation. To better understand the performance of a state-of-the-art system using this approach, we compare them to our proposed accelerator when solving CNF problems based on random 3-SAT benchmarking instances comprising 20 and 50 variables. We note that the SAT accelerators reported in the literature [11–15] are experimental proof-of-concept systems, whereas the goal of our work is to project the performance attainable by a fully integrated accelerator device. Compared to our simulations, these proof-of-concept systems employ larger technology nodes and potentially less-efficient memory technology. Moreover, proof-of-concept systems are typically operated in test environments that can produce additional energy overhead, for example, due to energy losses incurred from test circuit boards, cables, or external signal sources. We have therefore included both simulations using our energy model (based on the work reported in Ref. [10]) and a proof-of-concept system of the SAT accelerator reported in Ref. [11] that uses our IMC architecture to solve CNF problems.

Comparing the computational efficiency obtained from the simulations of our fully integrated accelerator and the proof-of-concept system in Ref. [11] shows that the fully integrated SAT accelerator can improve compute speed and energy efficiency by a factor of $\sim 5\times$ and $\sim 10\times$, respectively. Comparing the proof-of-concept system of our accelerator [11] to the one in Ref. [13], we find that the latter is similar in its capability, as it can solve k-SAT problems that have an arbitrary number of literals per clause using a WalkSAT-like heuristic. We find that our accelerator is $\sim 15\times$ faster and $\sim 2\times$ more energy efficient. Compared to the accelerators in Refs. [12, 14], we find that our accelerator is up to $\sim 2.4\times$ slower and up to $\sim 25\times$ less energy efficient in solving 3-SAT problems. It is important to note that these accelerators [12, 14] have been specifically optimized for solving 3-SAT problems, that is, problems with only 3 literals per clause. Because of this, whereas the accelerator in Ref. [13] could solve the benchmarking problems in Supplementary Figure 1 using the preprocessed representation (i.e., CNF-PP in Supplementary Figure 1), the

accelerators in Refs. [12, 14] would have to use the un-preprocessed problem representation (i.e., CNF in Supplementary Figure 1). As our data in Supplementary Figure 1 shows, these problems are larger in size and more complex to solve with SLS solvers, which results in an ∼30× increase in computation time and energy consumption. The benefits of the more energy-efficient 3-SAT solvers in Refs. [12, 14] would thus likely be offset by the overhead of mapping to a 3-SAT formulation. Based on our comparison, we estimate that our accelerator is either better than, or on-par with, other state-of-the-art accelerators when solving our benchmarking problems in CNF representation. Projecting the performance of solving the benchmarking problems in XNF representation from Supplementary Figure 1, an XNF-capable SAT accelerator would attain an additional improvement by an order of magnitude in both runtime and energy consumption over pure CNF SAT accelerators due to the decrease in problem size and the higher algorithmic efficiency. We also note that the number of clauses for our benchmarking instances in CNF representation can be prohibitively large to implement with many of the CNF SAT accelerators listed in Supplementary Table 3, whereas the XNF problems are sufficiently small to be implemented with existing crossbar arrays.

	VLSI 2024	ISSCC 2024	Sci. Rep. 2024	ISSCC 2023	VLSI 2025	This work
	experiment [12]	experiment [14]	experiment [15]	experiment [13]	experiment [11]	simulation based on Ref. [10]
Technology	65 nm CMOS	65 nm CMOS	65 nm CMOS	65 nm CMOS	55 nm CMOS	28 nm CMOS
Chip area (mm^2)	0.27	1.115	1.8	0.93	0.544	–
Max. order $k_{\max}$	3	3	2	128	64	–
Max. variables	20	50	20	128	64	–
Max. clauses	91	218	91	1024	256	–
Supported clauses						
CNF	yes	yes	no	yes	yes	yes
XNF	no	no	no	no	no	yes
Solution time 20/50 var. (μs)	7/NA	7/19	$16 \cdot 10^3$/NA	70/713*	5/45	1/10
Solution energy 20/50 var. (nJ)	11/NA	8/21	$15 \cdot 10^3$/NA	105/1098*	59/518	5/30

Supplementary Table 3: Comparison of SAT hardware accelerators for solving random 3-SAT problems comprising 20 and 50 variables. For the RRAM architecture simulations, the semi-analytical energy model does not place hard constraints on the number of variables or clauses. Chip area, $k_{\max}$, and maximum variables and clauses are thus not reported.
* Solution time and energy from Ref. [13] are reported for only 60 variables.

SUPPLEMENTARY NOTE 4: IMPACT OF ADC BIT RESOLUTION

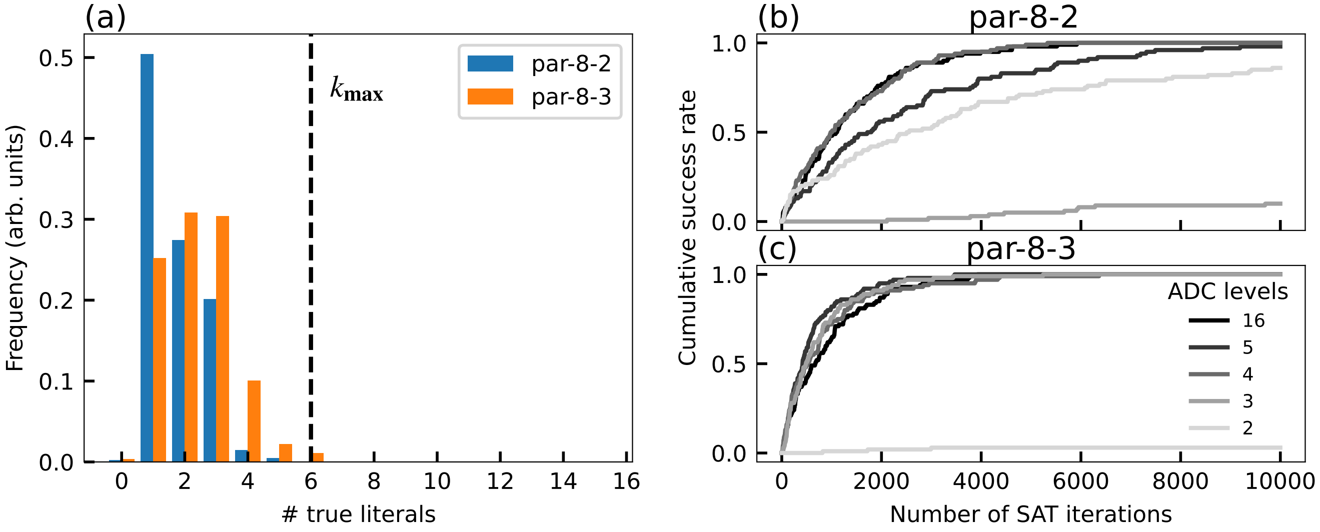

Supplementary Figure 4: **Effect of ADC bit resolution of solver performance** (a) Distribution of the number of true literals for the XOR clauses when solving the par-8-2 and par-8-3 instances. (b) and (c) Effect of the number of ADC levels on the cumulative success rate for the par-8-2 and par-8-3 instances, respectively.

In our architecture, accurate evaluation of XOR clauses requires a sufficiently high bit resolution of the ADC to distinguish between different numbers of true literals. Given that a problem has a maximum number of literals $k_{\max}$ in its XOR clauses, performing an accurate parity check can require up to $k_{\max}$ ADC levels. For that reason, the ADC resolution in our benchmarking experiment was chosen to be 4 bits to accommodate up to $k_{\max} = 15$ literals in the benchmarking problems. However, this bit resolution is required only in the case of all literals being true at the same time. When solving a problem, we find that this is rather unlikely to occur. In Supplementary Figure 4, we show the distribution of the true number of literals in the XOR clauses when solving the par-8-2 and par-8-3 instances. Both of these problems have up to $k_{\max} = 6$ literals. For the par-8-2 instance, we find that the average number of true literals is 1.7, while it is 2.4 for the par-8-3 instance. From the distribution, we can also see that detecting a higher number of true literals becomes increasingly unlikely.

The fact that the number of true literals can often be smaller than $k_{\max}$ opens up the possibility for optimizing the ADC bit resolution. Supplementary Figure 4b and Supplementary Figure 4c show the effect of reducing the ADC bit resolution on the cumulative success rate. For the par-8-2 instance, we find that the solver's performance is not significantly affected even with as few as 5 ADC levels. When further decreasing the bit resolution, we observe a decrease in the cumulative success rate. Notably, the problem can be solved with rather high probability even with just 2 ADC levels (effectively a comparator). For the par-8-3 instance, we observe that the success rate is barely affected by as few as 3 ADC levels. For 2 ADC levels, there is a significant drop in the success rate. These results indicate the potential to chose an ADC resolution lower than $k_{\max}$. As the power consumption of ADCs increases significantly with the bit resolution, employing fewer ADC levels would help further improve the energy efficiency of the XOR clause evaluation.

SUPPLEMENTARY NOTE 5: SENSITIVITY TO HARDWARE NON-IDEALITIES

As with any mixed-signal computing systems, the performance of analog computations for implementing WalkSAT-XNF is affected by device non-idealities. Such non-idealities can arise from device-to-device and cycle-to-cycle variations in the crossbar arrays and in the respective analog readout circuits. Device-to-device variations can be caused by process variations that occur during chip manufacturing, which can, for example, result in differences in the reference voltages supplied to components. As this variability is static after a chip has been fabricated, it can effectively be mitigated with calibration methods [16]. Device-to-device variations in RRAM cells, on the other hand, arise due to variations in the programmed conductance [17]. As shown in Fig. 3, conductance values after programming will typically be distributed around the target value with a standard deviation $\sigma_{\mathrm{d2d,RRAM}}$, leading to errors in the matrix representing the SAT problem. Compared to process variations, RRAM device-to-device variations cause changes to occur between each programming cycle, making error mitigation challenging. In addition, RRAM cells also create cycle-to-cycle variations due to thermal and telegraph noise [17]. Finally, cycle-to-cycle variations arise due to noise in the analog readout circuit (e.g., transimpedance amplifiers, comparators, ADCs) of each bit line (BL) within a crossbar array. Taking into account all these variations, the BL output voltage $V_{\mathrm{BL},i}$ during cycle t_k can be modelled by [16, 18]

$$V_{\mathrm{BL},i}(t_k) \propto \sum_j \left(G_{ij} + \mathcal{N}(0, \sigma_{\mathrm{d2d,RRAM}}) + \mathcal{N}(0, \sigma_{\mathrm{c2c,RRAM}})(t_k)\right) x_j + \mathcal{N}(0, \sigma_{\mathrm{c2c,readout}})(t_k) \ . \tag{1}$$

Here, x_j are Boolean variables, and G_{ij} are the target conductance values of the RRAM cells. Cycle-to-cycle variations of the RRAM cells and the readout circuit are approximated by a normal distribution $\mathcal{N}(\eta, \sigma)$ having a mean of $\eta = 0$ and a standard deviation of $\sigma_{\mathrm{c2c,RRAM}}$ and $\sigma_{\mathrm{c2c,readout}}$, respectively. The device-to-device variations of the RRAM cells after programming are approximated by a normal distribution with a mean of 0 and a standard deviation of $\sigma_{\mathrm{d2d,RRAM}}$. For device-to-device variations, samples are drawn from the normal distribution only once at the beginning of any simulation run. For cycle-to-cycle variations, samples are drawn at every cycle t_k.

To understand the impact of such device non-idealities on the performance of WalkSAT-XNF, we perform a simulation-based sensitivity analysis using Eq. (1). The conductance-dependent values for $\sigma_{\mathrm{c2c,RRAM}}$ and $\sigma_{\mathrm{d2d,RRAM}}$ are based on a model derived from experimental characterizations of TaO_x memristors [18, 20]. Here, we assume that the low resistance state (LRS, or ON state in Fig. 2) is set at 2 μS, whereas the high resistance state (HRS, or OFF state in Fig. 2) is set at 20 nS. For the analysis, we consider the effect of different levels of readout noise $\sigma_{\mathrm{c2c,readout}}$ on the performance of WalkSAT-XNF when solving the par8-1, par8-2, and par8-3 MDP benchmarking instances. Supplementary Figure 5a shows the cumulative success rates for solving the par8-3 instance under ideal conditions (i.e., $\sigma_{\mathrm{c2c,RRAM}} = \sigma_{\mathrm{d2d,RRAM}} = \sigma_{\mathrm{c2c,readout}} = 0$) as well as for different levels of readout noise $\sigma_{\mathrm{c2c,readout}}$, taking into account RRAM variations. Here, $\sigma_{\mathrm{c2c,readout}}$ has been normalized relative to the integer steps in $V_{\mathrm{BL},i}$. We find that a noise level of $\sigma_{\mathrm{c2c,readout}} = 0.15$ does not cause significant deviations from the ideal simulations, whereas increasing the noise further gradually decreases the success rate. Supplementary Figure 5b shows the ITS_{99} as a function of

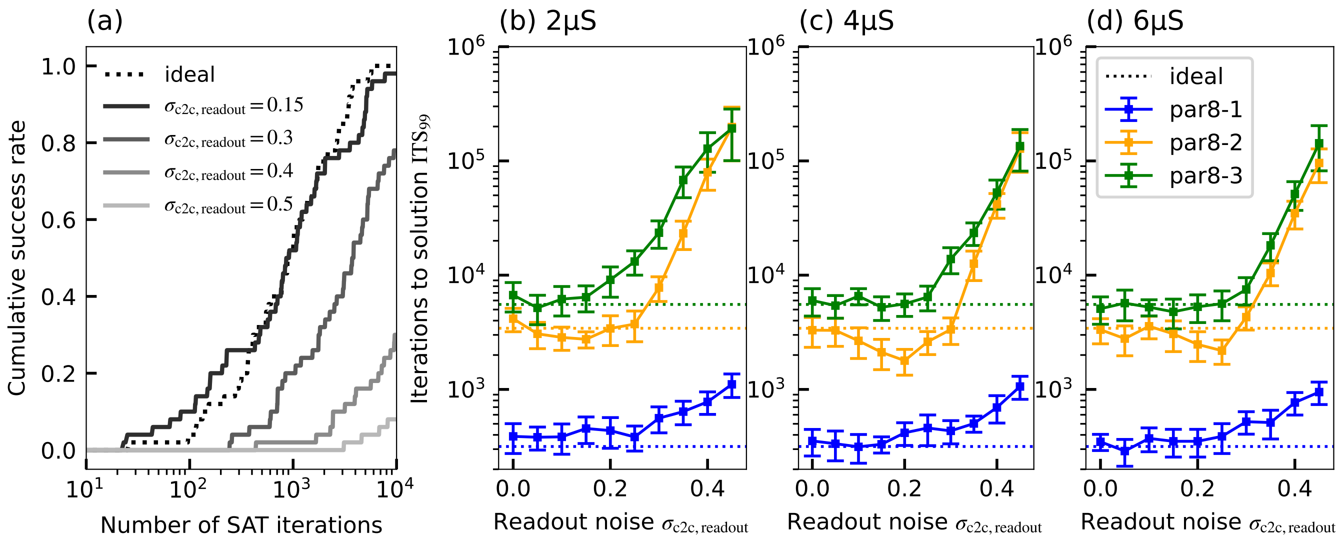

Supplementary Figure 5: **Sensitivity analysis of hardware non-idealities** (a) Comparison of the cumulative success rate of solving the par8-3 MDP benchmarking problem, for different levels of readout noise at an LRS conductance of 2 μS, as well as for the case (indicated by the dotted curve). (b–d) ITS_{99} as a function of the readout noise for an LRS conductance of 2 μS (b), 4 μS (c), and 6 μS (d). The error bars depict the standard error [19].

the readout noise. Compared to the ideal simulations (indicated using dotted lines), we find that the ITS_{99} does not increase significantly for noise levels of up to $\sigma_{c2c,readout} = 0.15$ approximately. Beyond that level, the ITS_{99} increases rapidly. Based on circuit simulations, we estimate that actual noise fluctuations in the readout circuit are in the range of just a few percent, and therefore well below the levels at which we observe a degradation in performance. This observation is also supported by the experimental data in Fig. 3, which shows that results obtained from ideal simulations agree well with results obtained from actual RRAM hardware.

We also analyze the robustness of ITS_{99} against noise in cases of higher LRS conductance. A higher LRS conductance increases the current flowing through the ON state RRAM cells relative to the OFF state cells, thereby enhancing the signal-to-noise ratio of the BL voltage $V_{BL,i}$. In Supplementary Figure 5c–d, we show the ITS_{99} for LRS at 4 μS and 6 μS. We find that increasing LRS conductance further enhances robustness, as the ITS_{99} does not increase significantly until a noise level of $\sigma_{c2c,readout} = 0.25$ approximately. While achieving improved robustness, the increase in current flow also increases the energy per iteration. For a conductance of 4 μS, we find that energy consumption increases by 5 percent for the MDP and AES classes and 3 percent for the McEliece class. For a conductance of 6 μS, the energy per iteration increases by 10 percent for the MDP and AES classes and 7 percent for the McEliece class. The fact that the LRS of the RRAM cells can be set to different conductance levels could thus be leveraged to further enhance the robustness of our architecture against non-idealities, with a relatively small energy overhead.

SUPPLEMENTARY REFERENCES

[1] J. M. Dudek, K. S. Meel, and M. Y. Vardi, The hard problems are almost everywhere for random cnf-xor formulas, in *Proceedings of the Twenty-Sixth International Joint Conference on Artificial Intelligence, IJCAI-17* (2017) pp. 600–606.

[2] W. Nawrocki, Z. Liu, A. Fröhlich, M. J. H. Heule, and A. Biere, Xor local search for boolean brent equations., in *SAT*, Lecture Notes in Computer Science, Vol. 12831, edited by C.-M. Li and F. Manyà (Springer, 2021) pp. 417–435.

[3] A. Biere, Yet another local search solver and Lingeling and friends entering the SAT Competition 2014, in *Proc. of SAT Competition 2014 – Solver and Benchmark Descriptions*, Department of Computer Science Series of Publications B, Vol. B-2014-2, edited by A. Balint, A. Belov, M. Heule, and M. Järvisalo (University of Helsinki, 2014) pp. 39–40.

[4] W. Nawrocki, Z. Liu, A. Fröhlich, M. J. Heule, and A. Biere, XOR local search for boolean brent equations, in *Theory and Applications of Satisfiability Testing–SAT 2021: 24th International Conference, Barcelona, Spain, July 5-9, 2021, Proceedings 24* (Springer, 2021) pp. 417–435.

[5] M. Soos, K. Nohl, and C. Castelluccia, Extending SAT solvers to cryptographic problems, in *Theory and Applications*

of Satisfiability Testing - SAT 2009, 12th International Conference, SAT 2009, Swansea, UK, June 30 - July 3, 2009. Proceedings, Lecture Notes in Computer Science, Vol. 5584, edited by O. Kullmann (Springer, 2009) pp. 244–257.

[6] M. Soos, S. Gocht, and K. S. Meel, Tinted, detached, and lazy cnf-xor solving and its applications to counting and sampling, in *International Conference on Computer Aided Verification* (Springer, 2020) pp. 463–484.

[7] M. Soos, J. Yang, S. Gocht, Y. Pote, K. S. Meel, and et al., Approxmc6: Approximate model counter, `https://github.com/meelgroup/approxmc` (2025), accessed: 2025-07-21.

[8] B. Andraschko, J. Danner, and M. Kreuzer, Sat solving using xor-or-and normal forms, Mathematics in Computer Science **18**, 1 (2024).

[9] M. Trimoska, S. Ionica, and G. Dequen, Parity (XOR) Reasoning for the Index Calculus Attack, in *Principles and Practice of Constraint Programming 2020* (Louvain-la-Neuve, Belgium, 2020) pp. 774–790.

[10] G. Pedretti, F. Böhm, T. Bhattacharya, A. Heittman, X. Zhang, M. Hizzani, G. Hutchinson, D. Kwon, J. Moon, E. Valiante, I. Rozada, C. E. Graves, J. Ignowski, M. Mohseni, J. P. Strachan, D. Strukov, R. Beausoleil, and T. V. Vaerenbergh, Solving boolean satisfiability problems with resistive content addressable memories, npj Unconventional Computing **2**, 7 (2025).

[11] T. Bhattacharya, D. Kwon, G. Hutchinson, X. Zhang, I. Rozada, and D. Strukov, A Fully Integrated Mixed-Signal Compute-In-Memory Accelerator for Solving Arbitrary Order Boolean Satisfiability Problems, in *2024 IEEE Symposium on VLSI Technology and Circuits (VLSI Technology and Circuits)* (2025).

[12] Q. Zhang, S. Su, Z. Liu, H.-C. Cheng, Z. Qiu, M. Palaria, J. Ye, D. Meng, B. Chen, S. Hossain, W. Wu, and M. S.-W. Chen, A stochastic analog sat solver in 65nm CMOS achieving $\mathbf{6.6\mu s}$ average solution time with 100% solvability for hard 3-sat problems, in *2024 IEEE Symposium on VLSI Technology and Circuits (VLSI Technology and Circuits)* (2024).

[13] S. Xie, M. Yang, S. A. Lanham, Y. Wang, M. Wang, S. Oruganti, and J. P. Kulkarni, 29.2 Snap-SAT: A One-Shot Energy-Performance-Aware All-Digital Compute-in-Memory Solver for Large-Scale Hard Boolean Satisfiability Problems, in *2023 IEEE International Solid- State Circuits Conference (ISSCC)* (IEEE, 2023) pp. 420–422.

[14] C. Shim, J. Bae, and B. Kim, 30.3 VIP-Sat: A Boolean Satisfiability Solver Featuring 5×12 Variable In-Memory Processing Elements with 98% Solvability for 50-Variables 218-Clauses 3-SAT Problems, in *2024 IEEE International Solid-State Circuits Conference (ISSCC)* (IEEE, 2024) pp. 486–488.

[15] H. Cilasun, Z. Zeng, S. Ramprasath, A. Kumar, H. Lo, W. Cho, W. Moy, C. Kim, U. Karpuzcu, and S. Sapatnekar, 3sat on an all-to-all-connected cmos ising solver chip, Scientific Reports **14**, 10757 (2023).

[16] A. Heittmann, M. Hizzani, and J. P. Strachan, Impact of variability compensation on the performance of an rram-based 3-sat solver, in *2025 IEEE International Symposium on Circuits and Systems (ISCAS)* (2025) pp. 1–5.

[17] G. Pedretti, E. Ambrosi, and D. Ielmini, Conductance variations and their impact on the precision of in-memory computing with resistive switching memory (rram), in *2021 IEEE International Reliability Physics Symposium (IRPS)* (2021) pp. 1–8.

[18] L. Zhao, L. Buonanno, A. Natarajan, J. Ignowski, and G. Pedretti, Noise aware finetuning for analog non-linear dot product engine, in *NeurIPS 2024 Workshop Machine Learning with new Compute Paradigms* (2024).

[19] M. Noori, E. Valiante, T. V. Vaerenbergh, M. Mohseni, and I. Rozada, A statistical analysis for per-instance evaluation of stochastic optimizers: How many repeats are enough? (2025), `https://arxiv.org/abs/2503.16589`, arXiv:2503.16589 [cs.LG].

[20] X. Sheng, C. E. Graves, S. Kumar, X. Li, B. Buchanan, L. Zheng, S. Lam, C. Li, and J. P. Strachan, Low-conductance and multilevel CMOS-integrated nanoscale oxide memristors, Advanced Electronic Materials **5**, 1800876 (2019), https://advanced.onlinelibrary.wiley.com/doi/pdf/10.1002/aelm.201800876.
